# Walking performance of persons with chronic stroke changed when looking down but not in dimly lit environment

**DOI:** 10.3389/fneur.2023.1186840

**Published:** 2023-06-15

**Authors:** Pei-Yun Lee, Yu-Chu Hseuh, Chih-Hung Chen, Sang-I Lin

**Affiliations:** ^1^Department of Physical Therapy, College of Medicine, National Cheng Kung University, Tainan, Taiwan; ^2^Department of Neurology, College of Medicine, National Cheng Kung University, Tainan, Taiwan; ^3^Institute of Long-Term Care, MacKay Medical College, New Taipei, Taiwan

**Keywords:** walking, vision, gait, stroke, environment

## Abstract

**Background and purpose:**

It is common to walk under different conditions, such as looking straight head, looking down at the feet or in dimly lit environment. The purpose of this study was to determine the impact of these different conditions on walking performance in persons with and without stroke.

**Methods:**

This was a case-control study. Persons with chronic unilateral stroke and age-matched control (*n* = 29 each) underwent visual acuity test, Mini Mental Status Examination (MMSE) and joint position sense test of the knee and ankle. The participants walked at their preferred speed under three walking conditions, looking ahead (AHD), looking down (DWN), and in dimly lit environment (DIM). A motion analysis system was used for the recording of the limb matching test and walking tasks.

**Results:**

Stroke participants differed from the control group in MMSE, but not in age, visual acuity or joint position sense. For the control group, the differences between the three walking conditions were nonsignificant. For the stroke group, DWN had significantly slower walking speed, greater step width and shorter single leg support phase, but not different symmetry index or COM location, compared to AHD. The differences between AHD and DIM were nonsignificant.

**Conclusion:**

Healthy adults did not change their gait patterns under the different walking conditions. Persons with chronic stroke walked more cautiously but not more symmetrically when looking down at the feet, but not in dimly lit environment. Ambulatory persons with stroke may need to be advised that looking down at the feet while walking could be more challenging.

## Introduction

Falling is a major problem for older adults and could lead to fear of falling, hospitalization, disability, lower quality of life and mortality (1, 2). For persons with stroke, the prevalence of falling is higher than the general older populations and most of the falls occur during walking and transfer (3, 4). Although approximately one to two thirds of persons with stroke eventually are able to walk independently, gait impairments, including slower speeds, instability, and asymmetry are common (5, 6), and have been found to be associated with increased risk of falling and fall-related injuries (7). Thus for persons with stroke, it is important to identify walking behaviors or situations that are more challenging and hence more likely to lead to loss of balance or falls.

One of the factors that may affect walking performance is visual conditions. Visual information is important for walking in several ways. When walking across flat obstacle-free terrains, a person typically looks ahead with the gaze focusing on the ground approximately two steps ahead (8, 9). For steering of locomotion, optic flow, a radial pattern of light produced at the eye of the participant when moving through the environment, and the perceived direction of a goal are particularly important (10–13). For vertical orientation and balance control in walking, visual, together with vestibular and somatosensory inputs, provide critical sensory information (14, 15). Visual information about the leg also has been found to improve the performance of healthy adults when foot placement needs to be better controlled, such as during obstacle crossing (16, 17), walking over uneven terrain (18), and ascending or descending stairs (19, 20). However, visual information about the leg is not necessary for appropriate foot placement when walking across flat obstacle-free terrains for healthy adults (21).

Compared to healthy adults, the role of visual information for the control of standing balance and upper limb reaching tasks has been found to be greater for persons with stroke, and these changes were not related to stroke location, or sensory impairments (22, 23). What is more, while visual concealment of the lower limbs had little effects on healthy participants during level walking, it significantly decreased the ability to attenuate trunk oscillation in persons with chronic stroke (24). Thus it seems that after stroke, visual inputs could play a greater role in the control of walking.

In daily living, one may need to walk under different visual conditions. For people whose movement and balance control rely more heavily on vision, changes in visual cues may affect walking performance, and possibly lead to increased risk of falls. The purpose of this study was to examine how different visual conditions for walking, including looking ahead, looking down at the feet and in dimly lit environment, affected walking performance in persons with and without stroke. It was hypothesized that, compared to looking straight ahead, gait symmetry would be improved in looking down because of increased visual cues about the lower limbs, and walking speed would be slower in dimly lit environment due to decreased visual cues in persons with stroke, but not in age-matched healthy adults.

## Methods

This was a cross-sectional case control study conducted in a gait laboratory.

### Participants

Twenty-nine persons with chronic stroke (onset >6 months) and 29 age-matched persons without stroke (control group) were recruited from the department of neurology of a tertiary hospital and nearby communities. The inclusion criteria for the stroke group were unilateral first time stroke with residual gait impairments, and able to follow experimental instructions and stand and walk for at least 10 m independently. Gait impairments were determined by a trained physical therapist experimenter by visual inspection. Participants who had unstable medical conditions or any neuromuscular or musculoskeletal conditions that could affect walking were excluded. For the control group, the inclusion criteria were no history of stroke or any current neuromuscular or musculoskeletal problems that could affect walking, and able to follow experimental commands. The Institutional Review Board of the BLINDED approved this study (number A-ER-104-382). All the research methods were performed in accordance with the provisions of the Declaration of Helsinki (as revised in Tokyo 2004). All the participants provided informed consents.

### Sensorimotor and cognitive function test

Participants underwent a modified Chinese version of the Mini Mental State Examination (MMSE, maximal score = 33) (25). Visual acuity was tested with a standard printed Snellen eye chart 6 m away with or without wearing a pair of shaded eye goggle. The side with better vision was used for data analysis. A limb matching task was used to measure the knee and ankle joint position sense. In this test, participants closed their eyes and sat in a customized height-adjustable chair with the thighs fully supported, lower legs dangling freely above the ground, and eyes closed. To test the knee joint position sense, the experimenter held the bilateral malleoli to move the affected lower leg approximately 15° into flexion or extension, stopped, and then instructed the participant to match the knee joint angle by moving the other lower leg without moving the rest of the body. To test the ankle joint position sense, the same initial position was adopted except that both heels were supported by a stool. The experimenter first held the first and fifth metatarsal heads to move the affected ankle joint into plantar- or dorsi-flexion, stopped, and then instructed the participant to match the ankle joint angle by moving the other foot without moving the rest of the body. Each task was repeated twice. The differences in the joint angles of the two sides of two repeated trials were used to represent joint position sense. For the stroke group, the Fugl-Meyer lower extremity assessment was used to assess the motor function of the affected lower limb (26).

### Walking tasks

The participants walked on an 8 meter paneled flat obstacle-free walkway in a motion lab under three conditions at their self-selected speeds, looking ahead (AHD), looking down at the feet (DWN), and in dimly lit environment (DIM) in random orders. The task performance was monitored by the experimenter using visual inspection to ensure that the task instructions were followed. A random order would be generated for each participant using the MS Excel to avoid sequence effects.

### Instrumentation and data reduction

An 8-camera SIMI motion analysis system (SIMI^®^ Reality Motion Systems GmbH, Unterschleissheim, Germany) was used for the recording of full body kinematics with a sampling rate of 100 Hz for the limb matching and walking tests. Twenty-six reflective markers were attached at specific anatomical landmarks to obtain the position data for the estimation of center of mass (COM) location, including the apex of the head, spinous process of C7, T8, and S2, and bilateral acromion of shoulder, lateral epicondyle of elbow, hand (midpoint between the ulnar and radial styloid process), anterior superior iliac spine (ASIS), greater trochanter, lateral and medial condyle of knee, lateral and medial malleolus of ankle, heel, and 2nd metatarsal head. Because the cameras were equipped with lights, thus shaded eye goggles were used to create a dimly lit environment.

Algorisms written in the Matlab (Version R2013a, The Math Works Inc. MA, United States) computer language were used to calculate the angle differences in the limb matching test, and stride characteristics and COM location in the walking tasks. For the calculation of joint position sense, the knee joint angle was defined as the angle between the thigh and shank segments, and the ankle joint angle as the angle between the shank and foot segments.

Before the data acquisition, each walking condition was practiced once. Immediately before the data acquisition, verbal instructions were given again. Each condition was performance once and the means of the two strides in the middle part of a walking trial were used for data analysis. To avoid inflating type I error due to conducting a large number of statistical analysis, this study examined the COM forward progression velocity to reflect walking velocity, and a few selected key stride characteristics, including step width, affected (dominant for the control group) side stride time and length and single limb support phase, and symmetry index of step time and length, and single limb support phase. Symmetry index was defined as the absolute value of 1 minus the affected side divided by the unaffected side (i.e., |1-(affected/unaffected)|). This definition was a modification from the symmetry ratio (affected/unaffected) recommended by a previous study for hemiplegic gait (27), and a greater value would be indicative of greater asymmetry.

For the estimation of COM, an 11-segment method was used, including foot, leg, thigh, forearm and hand, upper arm, and trunk head and neck segment (28). To estimate the standing posture during walking, the location of the COM relative the posterior margin of the base of support was calculated. It was defined as the averaged distance along the anteroposterior axis between the ground projection of the COM and the heel marker during the stance and swing phase. The affected and unaffected heel marker was used to represent the posterior margin of the base of support for the stance and swing phase, respectively.

### Statistical analysis

Between-group comparisons of the basic characteristics were conducted by independent t test for age, body mass index (BMI) and joint position sense, Mann–Whitney U test for MMSE and visual acuity, and *chi*-square for gender. For the within-group comparisons of the three walking conditions, four repeated measures multivariate analyses of variance (RMANOVAs) (29) were used for overall gait performance (COM velocity and step width), affected/dominant side stride characteristics (stride time and length, single limb support phase), symmetry index (step time and length, and single limb support phase), and COM location in relation to the base of support (stance, swing), separately for each group. For the RMANOVAs, the Mauchly’s test for the sphericity assumption was checked and most of the gait parameters did not violate the assumption (*p* > 0.05). For the purposes of statistical consistency, the RMANOVA was used for all the gait parameters. The LSD test (adjusted for multiple comparisons) was used for post-hoc analysis. The *p*-value was set at 0.05.

## Results

The basic characteristics of the study participants are shown in Table 1. The between-group differences in age, visual acuity with or without goggles and knee and ankle joint position sense were nonsignificant. The stroke group had significantly fewer females, greater BMI and lower MMSE.

**Table 1:** Basic characteristics of study participants.

	Control	Stroke	*p*
Age (year)	64.8 ± 8.3	61.6 ± 10.6	0.250
Gender (female)	20	10	0.018
BMI	22.8 ± 2.9	25.8 ± 4.2	0.002
MMSE	31.8 ± 1.1	29.4 ± 3.4	0.001
Visual acuity without goggle			
Mean/mode/median	0.77/0.8/0.8	0.72/1.0/0.7	0.884
Visual acuity with goggle			
Mean/mode/median	0.18/0.1/0.2	0.18/0.1/0.2	0.558
Joint position sense (°)			
Knee	4.37 ± 2.53	5.24 ± 3.86	0.377
Ankle	6.34 ± 3.01	7.27 ± 5.4	0.462
Stroke			
Time since onset (month)		41.5 ± 32.4	
Lesion type (*n*)			
Ischemic		25	
Hemorrhagic		4	
Lesion side (left)		14	
Location (*n*)			
Middle cerebral artery		2	
Posterior cerebral artery		1	
Frontal lobe		1	
Basal ganglion		8	
Internal capsule		3	
Brainstem		7	
Thalamus		4	
Lacunar		1	
Fugl-Meyer score of lower limb		21.6 ± 3.5	

The data are presented in mean ± standard deviation.

### Stride characteristics

**Figure 1:**
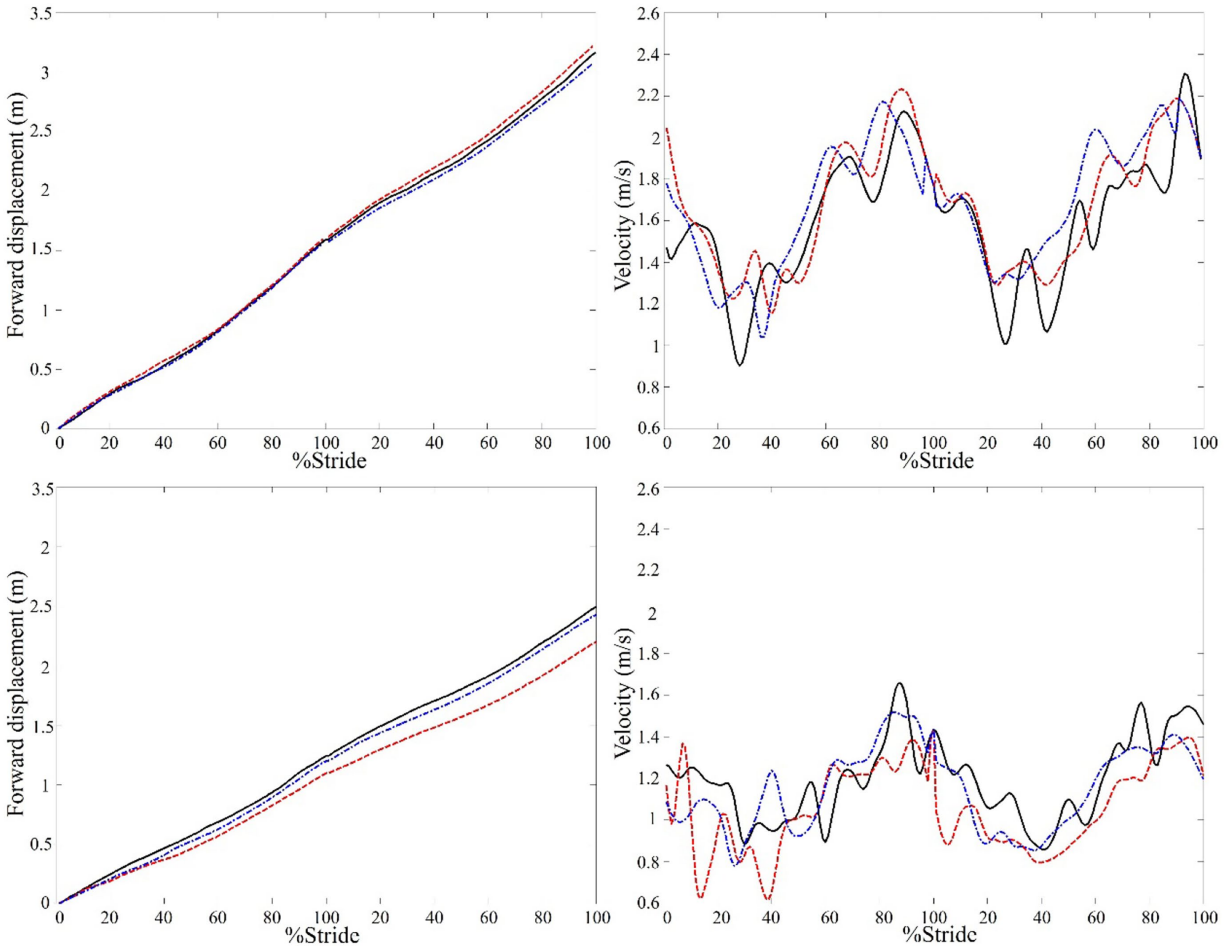
A representative example of the center of mass (COM) forward displacement and velocity of a control (top) and stroke participant with left hemiparesis (bottom) under three walking conditions. The data of two consecutive walking strides in looking ahead (solid line), looking down (dashed line), and dim (dashed dot line) conditions are shown. It can be seen that the COM displacement of the stroke participant was smaller and slower in looking down than looking ahead condition, whereas the changes in the control participant were small.

Figure 1 shows the forward progression and walking velocity of a representative control and stroke participant. For the overall gait performance (Table 2), the multivariate task effects were significant for the stroke group (Wilks’ lambda = 0.003) but not for the control group (Wilks’ lambda = 0.053). For the stroke group, univariate analysis showed that both walking speed and step width had significant task effects (*p* = 0.05 and 0.002, respectively) and follow-up paired-wise comparisons showed that DWN had significant slower walking speed (*p* = 0.038) and greater step width (*p* < 0.001) than AHD.

**Table 2:** Means and standard deviations of gait characteristics during different walking conditions.

	Control			Stroke		
	AHD	DWN	DIM	AHD	DWN	DIM
Overall performance^a^						
Gait velocity (m/s)^b^	1.48 ± 0.21	1.42 ± 0.25	1.50 ± 0.24	0.82 ± 0.45	0.77 ± 0.41	0.83 ± 0.46
Step width (m)^b^	0.09 ± 0.03	0.10 ± 0.04	0.09 ± 0.03	0.13 ± 0.04	0.15 ± 0.05	0.13 ± 0.05
Affected side stride characteristics^a^						
Stride length (m)	1.17 ± 0.13	1.13 ± 0.15	1.16 ± 0.13	0.84 ± 0.29	0.81 ± 0.28	0.82 ± 0.30
Stride time (sec)	1.02 ± 0.08	1.03 ± 0.11	1.01 ± 0.09	1.73 ± 1.07	1.85 ± 1.25	1.69 ± 1.02
Single limb support phase (% GC)^b^	37.8 ± 3.9	36.4 ± 3.1	37.4 ± 2.8	28.7 ± 1.0	26.8 ± 9.3	28.6 ± 1.0
Symmetry index						
Step length	0.16 ± 0.08	0.15 ± 0.09	0.16 ± 0.09	0.34 ± 0.34	0.39 ± 0.58	0.44 ± 0.54
Step time	0.09 ± 0.06	0.08 ± 0.05	0.09 ± 0.06	0.34 ± 0.39	0.39 ± 0.49	0.33 ± 0.37
Single limb support phase	0.12 ± 0.14	0.10 ± 0.06	0.08 ± 0.04	0.19 ± 0.15	0.22 ± 0.15	0.22 ± 0.20
Center of mass location (m)^a^						
Stance phase^c^	0.12 ± 0.07	0.10 ± 0.06	0.13 ± 0.09	0.13 ± 0.06	0.16 ± 0.06	0.11 ± 0.07
Swing phase^c^	0.28 ± 0.08	0.25 ± 0.07	0.28 ± 0.09	0.26 ± 0.08	0.27 ± 0.06	0.23 ± 0.08

AHD, looking straight ahead while walking; DWN, looking down at feet while walking; DIM, walking in dimly lit environment.

a For stroke group, *p* < 0.05 for multivariate analysis.

b For stroke group, *p* < 0.05 for univariate analysis between AHD and DWN.

c For stroke group, *p* < 0.05 for univariate analysis between DWN and DIM.

For the affected (dominant) side stride characteristics (Table 2), the multivariate task effects were significant for the stroke (Wilks’ lambda = 0.03) but not for the control group (Wilks’ lambda = 0.100). For the stroke group, univariate analysis showed significant differences in single limb support phase (*p* = 0.010) but not in stride length (*p* = 0.291) or stride time (*p* = 0.095), and follow-up paired-wise comparisons showed that DWN had significantly shorter single limb support phase than AHD and DIM (*p* = 0.006 and 0.007, respectively).

For symmetry index (Table 2), the multivariate task effects were nonsignificant for both stroke (Wilks’ lambda = 0.749) and control group (Wilks’ lambda = 0.390).

### COM location

For the location of COM in relation to the base of support (Table 2), the multivariate task effects were significant for the stroke (Wilks’ lambda = 0.008) but not for the control group (Wilks’ lambda = 0.537). For the stroke group, univariate analysis showed significant differences in both stance (*p* = 0.018) and swing phases (*p* = 0.047), and follow-up paired-wise comparisons showed that DWN had significantly greater distance than DIM in both the stance (*p* = 0.002) and swing (*p* = 0.009) phase.

## Discussion

In daily living, it is common to walking under different visual conditions. This study found that for persons with chronic stroke, looking down at the feet while walking was associated with cautious gait, i.e., slower walking speed, greater step width, and shorter single leg stance phase, compared to looking straight ahead. These changes were not accompanied by better gait symmetry and did not occur when walking in dimly lit environment or in age-matched healthy adults.

When walking in flat obstacle-free environments, an optimal combination of step length, width and duration would be selected to minimize energy cost (30, 31). When encountering increased task demands, such as greater balance threats, changes in walking pattern can be observed in healthy young and older adults (32, 33). These changes typically include slower walking speed, greater step width and shorter single leg stance time (34). It has been reported that slower walking speed primarily would be caused by shorter stride length in older adults (35, 36). Thus the changes in the stride characteristics in cautious gait are not likely to be the results of slower gait speed *per se*. In fact, this type of gait pattern is often adopted by persons with stroke in order to walk safely under the constraints of their sensorimotor and balance deficits (37, 38).

Looking down at the feet while walking is a common behavior and not necessarily associated with pathology. For healthy adults, this study found that such a behavior was not associated with significant changes in gait patterns, compared to looking straight ahead. These findings were expected because visual information about the leg is not necessary when walking in flat obstacle-free environments for healthy adults (8, 9, 21). For persons with stroke, however, looking down at the feet was found to be associated with significantly slower walking speed, and changes in stride characteristics, including greater step width and shorter single leg stance phase. The looking ahead and looking down conditions in this study could be different in several aspects. One was standing posture. In order to look down at the feet, a person might lean forward and move the body’s COM more anteriorly. A forward leaning standing posture has been found to be less stable (39), and could possibly result in cautious gait. This notion, however, was not supported by the finding that the posture for these two walking conditions, at least in terms of the location of the COM relative to posterior edge of the base of support, did not differ significantly.

Another difference was optic flow. Compared to looking ahead, the relative movement between the observer and the environment would be different in looking down, and thus optical flow would also be different. In a series of studies that manipulated optic flow, persons with stroke, but not controls, showed insufficient adaptation and errors in heading direction in response to changes in optic flow direction (40–42). This deficit could make looking down more challenging for persons with stroke. Further studies, possibly using virtual reality settings, are needed to determine how optic flow in looking ahead and looking down would affect locomotion control for persons with chronic stroke.

The attention demands for looking ahead and looking down might also be different. Looking down at the feet while walking involves an additional cognitive task, i.e., visual tracking. Adding a secondary task has been shown to interfere with balance and gait in healthy older adults (43, 44). Deterioration in dual-task walking performance has also been found in persons with stroke using secondary tasks that were more complexed than simple visual tracking (45, 46). It has also been found that the impact of a secondary task was greater in persons with stroke than age-matched healthy adults (45, 46). It is thus possible that persons with stroke, but not healthy adults, adopted cautious gait in looking down because of increased attention demand. To confirm the above notion, further studies are needed to determine if a simple visual tracking task would interfere with walking performance for persons with chronic stroke.

Walking in dimly lit environment is also a common but frown upon behavior for older adults because it may increase risk of falling. This study created a dimly lit environment which led to substantial reductions in visual acuity, although the participants were still able to see the walkway. In this condition, neither the stroke nor the healthy group markedly changed their walking pattern. It thus seemed that in flat, obstacle-free and familiar environment, darkness or poor visual acuity did not increase the task demand for walking for persons with chronic stroke or healthy older adults.

The findings of this study have important implications. Persons with stroke may need to be advised that while walking in flat and obstacle-free environments, looking down at the feet may make walking more difficult. For clinicians in stroke rehabilitation, different gaze directions or head postures may need to be included in gait training.

This study is limited in several ways. The stroke participants did not have apparent sensory impairments and were all ambulatory community-dwelling persons with chronic stroke. It is unclear if the findings could be generalized to persons with greater impairments or lower levels of functioning. Future studies that include those with more severe sensorimotor and ambulation problems are needed to provide a better understanding of the stroke population in general. Because of instrumentation limitations, the gaze was not monitored in this study, and thus whether the participants actually looked at the feet in DWN could not be ascertained. However, during the experiment, two experimenters would observe the task performances to ensure that the task instructions were followed. Most important, the instruction (looking down at the feet) was simple and easy to follow. Thus, this limitation was not likely to have a major impact on the findings. The participants were not instructed explicitly to modify their gait patterns based on the visual cues about the feet. Thus, the contribution of visual cues gained while looking down was not clear. Changes in the head position could lead to changes in vestibular inputs which could also affect walking. These factors were not controlled in this study and could affect the findings. However, they would not affect the interpretation that walking while looking down was associated with greater balance demands for stroke survivors. Further studies that monitor the gaze and head position and manipulate the instructions about the use of visual cues would be needed to clarify these issues. Due to insufficient sample size, between-group comparisons of task performances were not conducted, and thus limited the interpretation of the study findings. Future studies with larger sample sizes are needed to better demonstrate how the gait patterns of persons with stroke deviate from typical patterns under different conditions.

## Conclusion

For healthy adults, their gait patterns were not significantly affected by the different walking conditions. For persons with chronic stroke, looking down adversely affected their gait patterns, showing slower walking speed, larger step width and shorter single limb support phase. These changes were not accompanied by better gait symmetry and did not occur when walking in dimly lit environment. These findings suggested that looking down at the feet while walking could be more challenging. Persons with stroke may need to be advised to be cautious when looking down at the feet while walking or avoid such a behavior.

## Data availability statement

The raw data supporting the conclusions of this article will be made available by the authors, without undue reservation.

## Ethics statement

The studies involving human participants were reviewed and approved by Institutional Review Board, National Cheng Kung University Hosptial. The patients/participants provided their written informed consent to participate in this study.

## Author contributions

P-YL: conceptualization, methodology, data collection and analysis, project management, and manuscript writing. C-HC: conceptualization, methodology, and manuscript writing. Y-CH: methodology, data collection and analysis, and resources. S-IL: conceptualization, methodology, data collection and analysis, and manuscript writing. All authors contributed to the article and approved the submitted version.

## Funding

S-IL has received a research grant from the Ministry of Science and Technology, Taiwan (grant number: MOST 105-2314-B-006-011-MY3).

## Conflict of interest

The authors declare that the research was conducted in the absence of any commercial or financial relationships that could be construed as a potential conflict of interest.

## Publisher’s note

All claims expressed in this article are solely those of the authors and do not necessarily represent those of their affiliated organizations, or those of the publisher, the editors and the reviewers. Any product that may be evaluated in this article, or claim that may be made by its manufacturer, is not guaranteed or endorsed by the publisher.

## References

[1] Cumming RG Salkeld G Thomas M Szonyi G. Prospective study of the impact of fear of falling on activities of daily living, SF-36 scores, and nursing home admission. J Gerontol A Biol Sci Med Sci. (2000) 55:M299–305. doi: 10.1093/gerona/55.5.M299, PMID: 10819321

[2] Scuffham P Chaplin S Legood R. Incidence and costs of unintentional falls in older people in the United Kingdom. J Epidemiol Community Health. (2003) 57:740–4. doi: 10.1136/jech.57.9.740, PMID: 12933783 PMC1732578

[3] Weerdesteyn V de Niet M van Duijnhoven HJ Geurts AC. Falls in individuals with stroke. J Rehabil Res Dev. (2008) 45:1195–213. doi: 10.1682/JRRD.2007.09.0145 19235120

[4] Simpson LA Miller WC Eng JJ. Effect of stroke on fall rate, location and predictors: a prospective comparison of older adults with and without stroke. PLoS One. (2011) 6:e19431. doi: 10.1371/journal.pone.0019431, PMID: 21559367 PMC3084849

[5] Sheffler LR Chae J. Hemiparetic gait. Phys Med Rehabil Clin N Am. (2015) 26:611–23. doi: 10.1016/j.pmr.2015.06.006 26522901

[6] Olney SJ Richards C. Hemiparetic gait following stroke. Part I: characteristics. Gait Posture. (1996) 4:136–48. doi: 10.1016/0966-6362(96)01063-6

[7] Xu T Clemson L O'Loughlin K Lannin NA Dean C Koh G. Risk factors for falls in community stroke survivors: a systematic review and Meta-analysis. Arch Phys Med Rehabil. (2018) 99:563–573.e5. doi: 10.1016/j.apmr.2017.06.032, PMID: 28797618

[8] Foulsham T Walker E Kingstone A. The where, what and when of gaze allocation in the lab and the natural environment. Vis Res. (2011) 51:1920–31. doi: 10.1016/j.visres.2011.07.002, PMID: 21784095

[9] Patla AE. How is human gait controlled by vision. Ecol Psychol. (1998) 10:287–302. doi: 10.1080/10407413.1998.9652686

[10] Gibson JJ. The visual perception of objective motion and subjective movement. Psychol Rev. (1954, 1994) 101:318–23. doi: 10.1037/0033-295x.101.2.318 8022962

[11] Warren WH Jr Kay BA Zosh WD Duchon AP Sahuc S. Optic flow is used to control human walking. Nat Neurosci. (2001) 4:213–6. doi: 10.1038/84054 11175884

[12] Grasso R Prevost P Ivanenko YP Berthoz A. Eye-head coordination for the steering of locomotion in humans: an anticipatory synergy. Neurosci Lett. (1998) 253:115–8. doi: 10.1016/S0304-3940(98)00625-9, PMID: 9774163

[13] Sarre G Berard J Fung J Lamontagne A. Steering behaviour can be modulated by different optic flows during walking. Neurosci Lett. (2008) 436:96–101. doi: 10.1016/j.neulet.2008.02.049, PMID: 18400392

[14] Logan D Kiemel T Dominici N Cappellini G Ivanenko Y Lacquaniti F. The many roles of vision during walking. Exp Brain Res. (2010) 206:337–50. doi: 10.1007/s00221-010-2414-0, PMID: 20852990

[15] Wade MG Jones G. The role of vision and spatial orientation in the maintenance of posture. Phys Ther. (1997) 77:619–28. doi: 10.1093/ptj/77.6.619 9184687

[16] Marigold DS. Role of peripheral visual cues in online visual guidance of locomotion. Exerc Sport Sci Rev. (2008) 36:145–51. doi: 10.1097/JES.0b013e31817bff72, PMID: 18580295

[17] Patla AE Rietdyk S Martin C Prentice S. Locomotor patterns of the leading and the trailing limbs as solid and fragile obstacles are stepped over: some insights into the role of vision during locomotion. J Mot Behav. (1996) 28:35–47. doi: 10.1080/00222895.1996.9941731 12529222

[18] Marigold DS Patla AE. Visual information from the lower visual field is important for walking across multi-surface terrain. Exp Brain Res. (2008) 188:23–31. doi: 10.1007/s00221-008-1335-7, PMID: 18322679

[19] Miyasike-daSilva V Singer JC McIlroy WE. A role for the lower visual field information in stair climbing. Gait Posture. (2019) 70:162–7. doi: 10.1016/j.gaitpost.2019.02.033, PMID: 30875603

[20] Buckley JG Timmis MA Scally AJ Elliott DB. When is visual information used to control locomotion when descending a kerb? PLoS One. (2011) 6:e19079. doi: 10.1371/journal.pone.0019079, PMID: 21533113 PMC3078928

[21] Matthis JS Fajen BR. Visual control of foot placement when walking over complex terrain. J Exp Psychol Hum Percept Perform. (2014) 40:106–15. doi: 10.1037/a0033101, PMID: 23750964

[22] Bonan IV Colle FM Guichard JP Vicaut E Eisenfisz M Tran Ba Huy P. Reliance on visual information after stroke. Part I: balance on dynamic posturography. Arch Phys Med Rehabil. (2004) 85:268–73. doi: 10.1016/j.apmr.2003.06.017 14966712

[23] Meadmore KL Exell TA Burridge JH Hughes AM Freeman CT Benson V. Upper limb and eye movement coordination during reaching tasks in people with stroke. Disabil Rehabil. (2017) 40:2424–32. doi: 10.1080/09638288.2017.1336649 28597701

[24] Aoki O Otani Y Morishita S Domen K. The effects of various visual conditions on trunk control during ambulation in chronic post stroke patients. Gait Posture. (2017) 52:301–7. doi: 10.1016/j.gaitpost.2016.12.018, PMID: 28033576

[25] Folstein MF Folstein SE McHugh PR. Mini-mental state—a practical method for grading the cognitive state of patients for the clinician. J Psychiatr Res. (1975) 12:189–98. doi: 10.1016/0022-3956(75)90026-6 1202204

[26] Fugl-Meyer AR Jaasko L Leyman I Olsson S Steglind S. The post-stroke hemiplegic patient. 1. A method for evaluation of physical performance. Scand J Rehabil Med. (1975) 7:13–31. PMID: 1135616

[27] Patterson KK Gage WH Brooks D Black SE McIlroy WE. Evaluation of gait symmetry after stroke: a comparison of current methods and recommendations for standardization. Gait Posture. (2010) 31:241–6. doi: 10.1016/j.gaitpost.2009.10.014, PMID: 19932621

[28] Winter DA. Biomechanics and motor control of human movement. 4th *Edn.* Hoboken, NJ, USA: John Wiley & Sons (2009).

[29] Timm NH.2 Multivariate analysis of variance of repeated measurements, Handbook of Statistics, Vol. 1. Amsterdam, Netherland: Elsevier (1980), 41–87.

[30] Topp R Fahlman M Boardley D. Healthy aging: health promotion and disease prevention. Nurs Clin North Am. (2004) 39:411–22. doi: 10.1016/j.cnur.2004.01.007 15159189

[31] Minetti AE Capelli C Zamparo P di Prampero PE Saibene F. Effects of stride frequency on mechanical power and energy expenditure of walking. Med Sci Sports Exerc. (1995) 27:1194–202. doi: 10.1249/00005768-199508000-00014, PMID: 7476065

[32] Menant JC Steele JR Menz HB Munro BJ Lord SR. Effects of walking surfaces and footwear on temporo-spatial gait parameters in young and older people. Gait Posture. (2009) 29:392–7. doi: 10.1016/j.gaitpost.2008.10.057, PMID: 19041245

[33] Tsai YJ Lin SI. Older adults adopted more cautious gait patterns when walking in socks than barefoot. Gait Posture. (2013) 37:88–92. doi: 10.1016/j.gaitpost.2012.06.034, PMID: 22867560

[34] Maki BE. Gait changes in older adults: predictors of falls or indicators of fear. J Am Geriatr Soc. (1997) 45:313–20. doi: 10.1111/j.1532-5415.1997.tb00946.x 9063277

[35] Samson MM Crowe A de Vreede PL Dessens JA Duursma SA Verhaar HJ. Differences in gait parameters at a preferred walking speed in healthy subjects due to age, height and body weight. Aging. (2001) 13:16–21. doi: 10.1007/BF03351489 11292147

[36] Judge JO Davis RB III Ounpuu S. Step length reductions in advanced age: the role of ankle and hip kinetics. J Gerontol A. (1996) 51A:M303–12. doi: 10.1093/gerona/51a.6.m303 8914503

[37] Turnbull GI Wall JC. Long-term changes in hemiplegic gait. Gait Posture. (1995) 3:258–61. doi: 10.1016/0966-6362(96)82856-6

[38] Schinkel-Ivy A Wong JS Mansfield A. Balance confidence is related to features of balance and gait in individuals with chronic stroke. J Stroke Cerebrovasc Dis. (2017) 26:237–45. doi: 10.1016/j.jstrokecerebrovasdis.2016.07.022, PMID: 27955809 PMC5262475

[39] Lugade V Lin V Chou LS. Center of mass and base of support interaction during gait. Gait Posture. (2011) 33:406–11. doi: 10.1016/j.gaitpost.2010.12.013 21211977

[40] Lamontagne A Fung J. Gaze and postural reorientation in the control of locomotor steering after stroke. Neurorehabil Neural Repair. (2009) 23:256–66. doi: 10.1177/1545968308324549, PMID: 19060133

[41] Aburub AS Lamontagne A. Altered steering strategies for goal-directed locomotion in stroke. J Neuroeng Rehabil. (2013) 10:80. doi: 10.1186/1743-0003-10-80, PMID: 23875969 PMC3733933

[42] Lamontagne A Fung J McFadyen B Faubert J Paquette C. Stroke affects locomotor steering responses to changing optic flow directions. Neurorehabil Neural Repair. (2010) 24:457–68. doi: 10.1177/1545968309355985, PMID: 20067950

[43] Smith E Cusack T Cunningham C Blake C. The influence of a cognitive dual task on the gait parameters of healthy older adults: a systematic review and Meta-analysis. J Aging Phys Act. (2017) 25:671–86. doi: 10.1123/japa.2016-0265, PMID: 28253049

[44] Smith E Cusack T Blake C. The effect of a dual task on gait speed in community dwelling older adults: a systematic review and meta-analysis. Gait Posture. (2016) 44:250–8. doi: 10.1016/j.gaitpost.2015.12.017, PMID: 27004667

[45] Hyndman D Ashburn A Yardley L Stack E. Interference between balance, gait and cognitive task performance among people with stroke living in the community. Disabil Rehabil. (2006) 28:849–56. doi: 10.1080/09638280500534994, PMID: 16777772

[46] Al-Yahya E Dawes H Smith L Dennis A Howells K Cockburn J. Cognitive motor interference while walking: a systematic review and meta-analysis. Neurosci Biobehav Rev. (2011) 35:715–28. doi: 10.1016/j.neubiorev.2010.08.008, PMID: 20833198

